# From Preclinical Stroke Models to Humans: Polyphenols in the Prevention and Treatment of Stroke

**DOI:** 10.3390/nu13010085

**Published:** 2020-12-29

**Authors:** Edoardo Parrella, Cristina Gussago, Vanessa Porrini, Marina Benarese, Marina Pizzi

**Affiliations:** Division of Pharmacology, Department of Molecular and Translational Medicine, University of Brescia, 25123 Brescia, Italy; cristina.gussago@unibs.it (C.G.); v.porrini@unibs.it (V.P.); marina.benarese@unibs.it (M.B.); marina.pizzi@unibs.it (M.P.)

**Keywords:** polyphenols, flavonoids, stroke, brain ischemia, intracerebral hemorrhage, subarachnoid hemorrhage

## Abstract

Polyphenols are an important family of molecules of vegetal origin present in many medicinal and edible plants, which represent important alimentary sources in the human diet. Polyphenols are known for their beneficial health effects and have been investigated for their potential protective role against various pathologies, including cancer, brain dysfunctions, cardiovascular diseases and stroke. The prevention of stroke promoted by polyphenols relies mainly on their effect on cardio- and cerebrovascular systems. However, a growing body of evidence from preclinical models of stroke points out a neuroprotective role of these molecules. Notably, in many preclinical studies, the polyphenolic compounds were effective also when administered after the stroke onset, suggesting their possible use in promoting recovery of patients suffering from stroke. Here, we review the effects of the major polyphenols in cellular and in vivo models of both ischemic and hemorrhagic stroke in immature and adult brains. The results from human studies are also reported.

## 1. Introduction: The Burden of Stroke

Stroke is a cerebrovascular disease caused by the interruption of blood flow to the brain due to the blockage or rupture of a vessel and can affect both immature and mature brains.

Perinatal stroke occurs between the 20th week of gestation and the 28th day after birth with an incidence between 1/2300 and 1/5000 live births [1,2]. Perinatal stroke is not only a major cause of acute mortality in the early days of life, but newborn survivors may also develop neurological disabilities including cerebral palsy, cognitive deficits and behavioral disorders often lasting the entire lifetime [3,4].

Adult stroke is the second main cause of mortality and the third cause of disability worldwide [5,6]. Typical symptoms of stroke include understanding and speech issues; sudden unilateral weakness, numbness or loss of vision; ataxia; diplopia; dizziness; and nausea [5]. Moreover, stroke survivors face a significantly increased risk of developing depression and cognitive decline [7,8]. According to its etiology, stroke can be classified as ischemic, hemorrhagic or caused by subarachnoid hemorrhage [9,10,11]. Ischemic stroke, or brain ischemia, is the most frequent subtype of stroke, accounting for 85% of cases [9,10]. In ischemic stroke, the blood supply to part of the brain is reduced by the occlusion of a blood vessel either by an embolus or by local thrombosis [12]. About 10% of strokes are due to intracerebral hemorrhage (ICH), a severe neurological disorder associated with high rates of mortality and disability [13,14,15]. ICH results from the rupture of cerebral blood vessels that causes a rapidly expanding hematoma occurring within the brain parenchyma. Subarachnoid hemorrhage (SAH) accounts for approximately 5% of all strokes and is characterized by severe mortality and morbidity (more than 50%) [16,17]. In the major part of cases, SAH is caused by the rupture of an intracranial aneurysm [17].

Stroke in both immature and mature brains is a complex phenomenon that includes a series of pathological processes such as excitotoxicity, oxidative damage, apoptosis and inflammation, which eventually leads to cell death [18,19,20]. One of the main pathophysiological features of ischemic stroke is the brain–blood barrier (BBB) disruption, an event that occurs in almost two-thirds of patients in the first hours from the ischemia onset and causes vasogenic edema, hemorrhagic transformation and increased mortality [21,22]. Cerebral hemorrhage leads to a primary brain injury caused by increased intracranial pressure, followed by a secondary brain injury mediated by the physiological responses to hematoma, including inflammation [23].

Treatment options for stroke are currently very limited. The only approved pharmacological therapy for ischemic stroke is the recombinant tissue plasminogen activator (rtPA) [24,25]. Unfortunately, administration of rtPA after 4.5 h from the ischemic event is contraindicated for the risk of hemorrhagic conversion, limiting the use of this drug [25]. Neurosurgical interventions are performed to remove blood in ICH [26] or to treat brain aneurysm in SAH [27].

In light of the above considerations, there is an urgent need for the development of new therapies able to prevent or reduce stroke neuronal injury.

## 2. Polyphenols: Definition and Classification

Polyphenols are molecules chemically characterized by the presence of at least one aromatic ring with one or more hydroxyl groups attached [28,29]. Polyphenols are plant secondary metabolites that are thought to help plants to survive and proliferate, protecting them against microbial infections or herbivorous animals, or luring pollinators [30]. Polyphenols are found in many medicinal and edible plants which represent important alimentary sources, including fruits, vegetables, beverages (such as tea and red wine) and extra virgin oil [31].

**Figure 1:**
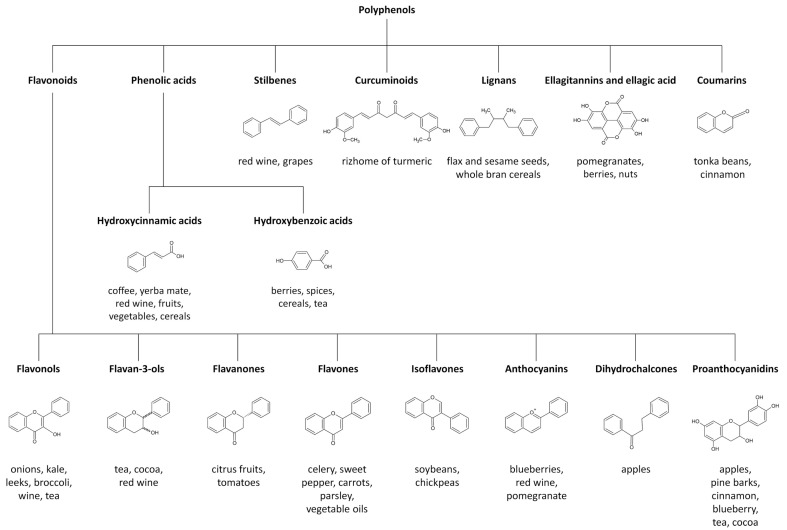
Major polyphenol classes and their most important food sources.

This group of natural products includes a broad number of different compounds, ranging from simple molecules with low molecular weight to complex and large derived polyphenols [28,29]. According to their chemical structure, polyphenols can be classified into various classes including flavonoids, phenolic acids, stilbenes, curcuminoids, lignans, ellagitannins and ellagic acid and coumarins [28,29]. Flavonoids are structurally based on a skeleton of fifteen carbons, with two aromatic rings connected by a three-carbon bridge. They are the most numerous of polyphenols and are widely distributed through the plant kingdom [28,29]. The main subclasses of dietary flavonoids include flavonols, flavan-3-ols, flavanones, flavones, isoflavones, anthocyanins, dihydrochalcones and proanthocyanidins [28,29]. Among the non-flavonoid polyphenols, phenolic acids can be further divided into hydroxycinnamic acids and hydroxybenzoic acids [28,29]. Figure 1 depicts the classification of polyphenols and describes some known food sources for each molecular class.

Among the many micronutrients present in plants, polyphenols are the most numerous and particularly endowed with beneficial properties [32]. For these reasons, polyphenols have been widely investigated for the prevention and treatment of several pathological conditions, including cancer, neurodegenerative disorders, metabolic and cardiovascular diseases and stroke [33,34,35].

## 3. Polyphenol Metabolism: Role of Gut Microbiota

Polyphenol bioavailability is generally poor, and only 1–10% of total polyphenol intake is detectable in blood and urine samples [33]. Bioavailability is particularly low for flavones, stilbenes and curcumin and is slightly higher for tea flavan-3-ols, flavanones in citrus fruits, soy isoflavones and red wine anthocyanidins [36,37,38]. However, maximum polyphenol concentration in plasma remains extremely low and rarely exceeds 1 μM, even in individuals consuming a polyphenol-rich diet [36]. How polyphenols exert their beneficial actions despite their poor bioavailability is not clear yet. A possible explanation may rely on the fact that many polyphenol metabolites exhibit a biological activity [39].

Polyphenols are generally consumed with the diet or as supplements. A proportion of ingested polyphenolic compounds can be absorbed in the small intestine and metabolized by phase II enzymes. However, the major part of polyphenols reach the large intestine where they are degraded by intestinal microbiota. A large body of evidence indicates a fundamental role of colonic microorganisms in determining the bioavailability and activity of polyphenols by transforming them into readily absorbable molecules or biologically active metabolites [32,40,41]. The relationship between polyphenols and microbiota is bidirectional and, if the intestinal bacteria modulate polyphenol metabolism, polyphenolic compounds can in their turn influence the composition of the microbial population [32,42].

Different findings suggest the gut microbiota could modulate the activity of polyphenols potentially active against stroke. For example, flavan-3-ols, phenolic compounds characterized by a generally low bioavailability, are extensively metabolized by host and gut microbiota enzymes. Phenyl-γ-valerolactones and phenylvaleric acids, the main microbial metabolites of flavan-3-ols, might be responsible for the beneficial effects attributed to their parent compounds, including neuroprotection [43]. Daidzein, an isoflavone endowed with beneficial properties enriched in soy food, is metabolized by gut microbiota to equol, which possesses higher antioxidant activity and affinity for estrogen receptors than the parent compound. The neuroprotective flavone glycoside baicalin (baicalein 7-O-glucuronide) can only be absorbed after hydrolysis by gut microbiota β-glucuronidase to the aglycone form baicalein [44]. Similarly, the neuroprotective anthocyanin cyanidin-3-O-glucoside displays a poor bioavailability, while its microbiota degradation products are more easily absorbable [45]. Ellagic acid and ellagitannins are degraded by intestinal microorganisms to form urolithins, molecules characterized by higher bioavailability and better anti-inflammatory and antioxidant properties than their compounds of origin [46].

## 4. Polyphenols: Mechanisms of Action

The major mechanism of natural polyphenols in preventing stroke relies on their protective action on the cardiovascular system [34,47,48].

Many polyphenols are endowed with anticoagulant and antiplatelet activities, potentially contributing to the prevention of thrombus formation, the main cause of ischemic stroke [49,50]. For example, several coumarin derivatives exert anticoagulant properties by inhibiting the vitamin K epoxide reductase complex and are widely used as clinical anticoagulant agents [51]. Among the polyphenols endowed with antiplatelet activity, the isoflavones genistein and daidzein possess a marked and physiologically relevant cyclooxygenase-1 (COX-1) inhibitory activity [52]. Other flavonoids with antiaggregant effects, including the isoflavone tectorigenin, have been reported to act as antagonists on thromboxane receptors [52,53].

Hypertension, a long-term medical condition affecting millions of individuals worldwide [54], is an important risk factor in particular for ICH and SAH subtypes of stroke [55,56]. Dietary intake of flavonoids belonging to anthocyanin, flavone and flavan-3-ol subclasses may contribute to the prevention of hypertension [57,58]. The underlying biological mechanisms by which polyphenols regulate blood pressure include vasodilation through the regulation of nitric oxide (NO) and endothelium-derived hyperpolarizing factor (EDHF) [47,57,58].

Besides the well-documented beneficial effects of polyphenols on cardio- and cerebrovascular systems, a growing number of studies in cellular and animal stroke models indicates a direct protective effect of many polyphenols on the brain. Notably, several polyphenols exert neuroprotective actions in preclinical models even when administered after stroke induction, indicating that these molecules may be useful not only in increasing resilience to brain damage, but also for the recovery of patients suffering from stroke. Moreover, the fact that different polyphenolic compounds act on the same molecular pathways raises the possibility that they may promote synergistic effects at very low doses. Therefore, the possible synergistic effect between polyphenols with each other or with other compounds may provide the rationale to overcome the limitations caused by the poor bioavailability of these molecules.

At the mechanistic level, polyphenols exert their neuroprotective benefits by acting on several targets simultaneously. These compounds are generally strong antioxidants, working as reactive oxygen species (ROS) scavengers and metal chelators due to the presence of hydroxyl groups and neutrophilic centers [33]. Furthermore, many polyphenols are able to activate transcription factors involved in antioxidant-responsive element pathways, such as erythroid 2-related factor 2 (Nrf2), thus promoting the expression of antioxidant enzymes including superoxide dismutase (SOD), heme oxygenase-1 (HO-1), catalase, glutathione reductase and glutathione-S-transferase [59].

Apoptosis is a process that can play a primary role in various pathologies, including cardiovascular diseases and stroke [60]. Many polyphenols are able to interact with proteins regulating apoptosis, including proapoptotic (Bax, Bad) and antiapoptotic (Bcl-2, Bcl-XL) members of Bcl-2 family, p53, mitogen-activated protein kinases (MAPKs) and protein kinase B (AKT) [60]. These compounds can act as pro- or antiapoptotic agents, depending on their concentrations, cellular system and stage of pathological process [60].

The polyphenol-mediated neuroprotection not only involves a direct effect on neurons, but also modulatory effects on different inflammation players in the brain, including microglia and mast cells (MCs) [61,62]. The anti-inflammatory properties of polyphenols are based on their capability to interfere with immune cell regulation, inflammatory gene expression and the synthesis of inflammatory mediators [63]. For example, a number of polyphenols have been shown to modulate nuclear factor kappa-light-chain-enhancer of activated B cells (NF-kB), toll-like receptor (TLR) and arachidonic acid pathways, suppressing the production of tumor necrosis factor α (TNF-α), interleukin (IL)-1β, IL-6, IL-1 and IL-8, as well as cyclooxygenase-2 (COX-2), inducible nitric oxide synthase (iNOS) and nitric oxide (NO) [63].

Epigenetic modifications, including DNA methylation, histone modifications and RNA-based mechanisms, modify gene expression without altering the DNA sequence. Epigenetic modifications regulate important physiological processes in living organisms, but they have also been associated with the pathogenesis of various diseases, including stroke [64]. Various polyphenols can influence epigenetic mechanisms underlying stroke pathogenesis and progression by modulating DNA methylation and histone modifications through the interaction with histone deacetylases (HDACs) and DNA methyltransferases (DNMTs) [65].

The hypothesized mechanisms of action of individual polyphenols active in models of stroke are discussed in the following section.

## 5. Polyphenols and Stroke: Results from Preclinical Stroke Models

Isolated polyphenols active in preclinical stroke models are described below. In this review, polyphenols have been clustered according to their chemical structure. The major part of polyphenols exists in plants as glycosides, where different sugars are bonded to the polyphenolic structure in different positions. For simplicity, we have classified polyphenols according to the chemical structures of the aglycones.

### 5.1. Flavonoids

#### 5.1.1. Flavonols

Flavonols, the most ubiquitous class of flavonoids in foods, display antioxidant and anti-inflammatory properties [66]. They are present mainly in onions, kale, leeks and broccoli, but also in red wine, tea and fruits.

Quercetin is a plant flavonol widely distributed in nature; common sources of quercetin are red onions and kale. The molecule is a strong antioxidant and anti-inflammatory agent and displays potential protective properties against hypertension and ischemic heart disease in both animal models and humans [67,68]. Notably, quercetin and its glycosides isoquercetin (3-O-glucoside) and rutin (3-O-rutinoside) have been found to promote beneficial effects in various models of brain ischemia [69,70,71,72,73,74], ICH [75,76] and SAH [77,78]. The health effect of quercetin could be attributed to its antioxidant, antiapoptotic and anti-inflammatory actions, combined with a protective effect on BBB through the inhibition of metalloproteinase (MMP) activity [70,72,73,74,75,76,77]. It has been proposed that the antioxidant and antiapoptotic activity of the molecule may be mediated by the activation of Nrf2 factor [69].

Myricetin can be found in various medicinal herbs, vegetables (tomatoes) and fruits. The molecule has been extensively studied for its multiple pharmacological activities, including antiapoptotic, anti-inflammatory and antioxidant properties [79]. Studies have revealed that myricetin acts against ischemic damage in both oxygen–glucose deprivation (OGD) cellular model [80] and in rats subjected to transient middle cerebral artery occlusion (tMCAO) [80,81]. Moreover, myricetin reduced endothelial permeability in human brain microvascular endothelial cells (BMVECs) subjected to OGD, suggesting a beneficial role in maintaining BBB function [82]. Among the mechanisms proposed to explain myricetin-induced protection, there are the inhibition of p38 MAPK and the activation of AKT and Nrf2 factors [80,81,82].

Kaempferol is present in a variety of plants and fruits including *Carthamus tinctorius* (safflower), beans and broccoli [83]. A protective effect of kaempferol or its glycosides (kaempferol-3-O-rutinoside and kaempferol-3-O-glucoside) has been shown in tMCAO rats [84,85,86]. These studies suggest that postischemic treatment with kaempferol prevents neuroinflammation by decreasing activation of NF-kB/RelA and STAT3 [85,86].

Fisetin is a flavonol found in various fruits and vegetables, including apple, strawberry, persimmon and onion. The molecule has been reported to exert several beneficial effects, including neuroprotective activities [87]. For example, fisetin improved outcomes in the rabbit small clot embolism (SCE) model [88] or in rats subjected to permanent middle cerebral artery occlusion (pMCAO) [71] when administered the first minutes following ischemia. Notably, treatment of tMCAO mice with fisetin even 3 h after ischemia reduced infarct size and immune cell activation and infiltration [89].

Morin, a natural flavonol found in the branches of *Morus alba* (white mulberry) and other Chinese medicine plants, exhibits a wide spectrum of antioxidant and antiapoptotic activities [90]. Pre- and poststroke treatment with morin ameliorated brain damage, BBB leakage and neurological deficits in tMCAO rats by reducing oxidative stress, apoptosis and inflammation [91,92].

#### 5.1.2. Flavan-3-ols

Flavan-3-ols are particularly abundant in tea plants, as well as in cocoas and chocolates. These polyphenols are considered primarily responsible for tea-promoted health benefits thanks to their cardioprotective and neuroprotective activities [93,94]. Flavan-3-ols are effective also in protecting against ischemic insults, as suggested by studies on green-tea-based supplements in preclinical models of stroke [95,96,97]. There are also numerous pieces of evidence indicating an antistroke activity for individual flavan-3-ols.

Epigallocatechin-3-gallate (EGCG), the predominant and most studied flavan-3-ol in green tea, induced preconditioning against OGD in a cellular model of brain ischemia [97]. EGCG has been shown to ameliorate cerebral damage and neurological deficits in different rodent species subjected to brain ischemia [98,99,100,101,102,103,104,105,106]. Notably, coadministration of EGCG with rtPA reduced the side effects of delayed rtPA treatment in a rat tMACO model, suggesting a potential clinical use of EGCG as an adjuvant in stroke therapies [107]. Multiple mechanisms have been proposed as mediating the protective effect of the molecule, including suppression of MMP activation [98], antioxidant effects [100,103,104] (possibly through activation of Nrf2 [104]), attenuation of inflammation [102] and reduction of apoptosis via modulation of phosphoinositide 3-kinase (PI3K)/Akt signaling [103]. Moreover, recent findings pointed out a beneficial effect of EGCG in cellular and animal models of SAH by targeting hemoglobin (Hb)-induced mitochondrial dysfunction [108,109,110].

The chemical structure of (-)-epicatechin-3-gallate (ECG) is similar to that of EGCG. The protective effect of ECG on cells subjected to OGD was consistent with EGCG [97]. Moreover, ECG protected human BMVECs against ischemic insult by promoting neovascularization and modulating apoptosis and autophagy [111].

(-)-Epicatechin (EC) is particularly abundant in cocoa, dark chocolate and green tea. The molecule exerted protective effects in cellular and animal models of brain ischemia, namely OGD-injured neurons and rats subjected to MCAO [112,113]. Similarly, EC was effective in ICH models, protecting cultured astrocytes against Hb-induced damage and mice from intracerebral hemorrhage [114,115]. The EC protection could be partly mediated by the activation of Nrf2 signaling [112,113,114,115].

#### 5.1.3. Flavanones

Flavanones are present in citrus fruits and tomatoes and their juices. Compared to other flavonoids, flavanones show a weaker radical scavenging activity. However, these molecules can reduce oxidative stress by targeting the Nrf2/HO-1 axis [116]. The antioxidant and anti-inflammatory properties of the flavanones suggest a potential use of these molecules for the prevention or treatment of cardiovascular diseases [117]. Various studies also support a role of flavanones against stroke.

Eriodictyol is a compound isolated from the Chinese herb *Dracocephalum rupestre* and citrus fruits [118]. The treatment of mice with eriodictyol prevented neuronal death, reduced infarct area and neuroinflammation and improved neurological and memory deficits induced by ischemic insult [119]. Eriodictyol-7-O-glucoside was effective in protecting astrocytes against OGD-induced ischemia and in reducing brain damage and neurological deficits in rats subjected to tMCAO by targeting Nrf2 signaling [120].

Naringenin is a natural flavanone endowed with neuroprotective properties [121]. The molecule protected culture neurons against hypoxic injury by reducing oxidative stress and mitochondrial dysfunction via activation of Nrf2 signaling [122,123]. Interestingly, naringenin nanoparticles rescued human mesenchymal stem cells (MSCs) from OGD-mediated stress, suggesting a potential use of the molecule as a strategy to improve MSC-based strategy against stroke [124]. Naringenin also showed a neuroprotective profile in an animal model of brain ischemia, reducing apoptosis, inflammation, oxidative stress and neurological deficits through the modulation of claudin-5, MMP9, Nrf2, nucleotide oligomerization domain-like receptor 2 (NOD2) and NF-κB [123,125,126]. Finally, diet supplementation of naringin, the naringenin-7-O-glycoside, prevented cerebral thrombogenesis in pial microvessels of stroke-prone spontaneously hypertensive rats [127].

Hesperetin and its glycoside hesperidin (hesperetin-7-O-glycoside) can be isolated from the rinds of some citrus species. Preclinical studies have shown the potential of these molecules in the treatment of neurological and cardiovascular pathologies [128]. Pretreatment with hesperetin ameliorated functional and histological outcomes in an MCAO rat model [129]. Similarly, hesperidin nanoparticles reduced infarct volume, inflammatory cytokines and oxidative stress in rats subjected to two-vessel occlusion (2VO, also called bilateral common carotid artery occlusion (BCCAO)) [130]. As observed for naringenin, hesperidin consumption through the diet reduced thrombotic tendency in stroke-prone spontaneously hypertensive rats [127], raising the possibility that daily ingestion of these flavonoids could promote an antithrombotic effect. Hesperidin could also be useful in treating cerebral vasospasm, as recently suggested by its beneficial effects on vessel walls and luminal diameters in an SAH rat model [131].

In recent years, there has been a growing interest in the neuroprotective agent pinocembrin, a flavanone particularly abundant in propolis [132]. Pinocembrin promoted protection against ischemic stress in neurons, BMVECs or in a cellular BBB model subjected to OGD [133,134,135]. The compound was effective also in different animal models of brain ischemia [134,136,137,138]. Notably, pinocembrin pretreatment extended the therapeutic time window of rtPA treatment in a rat model of brain ischemia [135]. Possible mechanisms for pinocembrin activity in preclinical models of brain ischemia include inhibition of MMPs, BBB protection, autophagy modulation, inhibition of apoptosis and inflammatory cascade [133,134,135,136,137,138]. Additionally, pinocembrin improved early outcomes in an ICH mouse model by inhibiting toll-like receptor 4 (TLR4) and modulating microglia activation [139].

#### 5.1.4. Flavones

Flavones are a class of polyphenols widely distributed in the plant kingdom, including several vegetables and fruits that are components of the human diet [140]. Different flavones have been explored for neuroprotection in preclinical models.

Luteolin is a flavone found in different vegetables, fruits and medicinal herbs including celery, carrots and broccoli [141]. Luteolin and its glycosides orientin (8-C-glucoside) and luteoside (7-O-glucoside) have been reported to exert beneficial effects in cellular and animal models of neonatal hypoxic–ischemic brain injury (NHIBI) [142], brain ischemia [143,144,145,146,147] and ICH [148,149]. The molecule engages several mechanisms of action that could play a role in its antistroke action, including antiapoptotic activity, BBB stabilization by claudin-5 upregulation and MMP9 inhibition, reduction of oxidative stress and autophagy enhancement through the activation of Nr2 pathway, MCs modulation, reduction of inflammation via activation of PPARγ and downregulation of TLR4/NF-κB pathway [144,145,146,147,148,149].

Apigenin is a natural flavone present in vegetables and fruits such as celery, parsley, tea, onion and grapefruit [150]. Recent findings suggested that the molecule could also alleviate brain damage and ameliorate poststroke neurological and cognitive deficits in brain ischemia [151,152] and SAH [153] models. Apigenin protective effects seem to rely on multiple mechanisms involving the promotion of angiogenesis via caveolin-1/vascular endothelial growth factor (VEGF) pathway and reduction of TLR4-mediated inflammation [151,153,154]. The effect of apigenin in alleviating poststroke cognitive impairment was found to involve the epigenetic induction of brain-derived neurotrophic factor (BDNF) through HDAC inhibition [152].

Nobiletin is a flavone extracted from the peel of citrus fruits. The molecule is endowed with several beneficial properties, including neuroprotective activities [155]. Nobiletin also exerted neuroprotective action in animal models of stroke, reducing cerebral apoptosis and inflammation and improving learning and memory deficits following ischemia [156,157].

Tangeretin is a citrus flavone endowed with neuroprotective properties [158]. The molecule was able to protect against OGD insult by preventing activation of proapoptotic c-Jun N-terminal kinase (JNK) signaling [159]. Both nobiletin and tangeretin improved the viability of the human hepatocellular carcinoma cells (HepG2) under hypoxic conditions [160]. The two molecules have been identified as the active compounds in the extract of *Pericarpium aurantii*, the immature fruit of *Citrus aurantium*, able to attenuate brain pathology in tMCAO rats [160].

Baicalein and its glycoside baicalin are flavones extracted from *Scutellaria baicalensis*, a traditional Chinese herb. Over the years, a growing body of evidence has pointed out the neuroprotective and antistroke activities of these molecules [161,162]. Baicalin reduced OGD-mediated neurotoxicity in the human neuroblastoma cell line SH-SY5Y [163]. Injected after the onset of stroke, this molecule reduced infarct size and improved neurological deficits in tMCAO rats [163,164]. Additionally, poststroke baicalin administration alleviated brain damage in a gerbil model of global cerebral ischemia by antioxidative and antiapoptotic mechanisms [165].

Acacetin is a naturally occurring flavone known for its numerous pharmacological activities, including neuroprotective and anti-inflammatory properties [166]. Recent findings indicate that acacetin administration to mice subjected to tMCAO promoted neuroprotection, possibly via inhibition of microglial activation and Nod-like receptor family, pyrin domain containing 3 (NLRP3) inflammatory signaling [167].

#### 5.1.5. Isoflavones

Isoflavones are found primarily in legumes, including soybeans and chickpeas, but also in fruits such as raisins and currants. In the human diet, the main sources of isoflavones are soybeans and soy food, which contain mainly genistein and daidzein [168,169]. For their chemical properties, isoflavones have been investigated as a therapy for cardiovascular and cerebrovascular pathologies [168,169].

A variety of preclinical studies support a beneficial role of the isoflavone genistein in protecting the brain against stroke (for complete reviews, see [170,171]). Briefly, the molecule promoted neuroprotection in cellular cultures subjected to OGD [172,173], in global and focal in vivo models of brain ischemia [174,175,176,177,178,179,180,181,182,183,184,185,186,187] and in animal models of SAH [188,189]. Genistein engages several mechanisms of action, including reduction of oxidative stress, inflammation and apoptosis through the promotion of antiapoptotic and growth factors such as Bcl2 and Nrf2 [170,171].

The isoflavone daidzein reduced injury and/or enhanced functional recovery in rats or mice subjected to MCAO when administered not only before but also after stroke onset [190,191,192]. It has been suggested that the beneficial effect of the molecule could rely on its capability to decrease oxygen free radical production [192] and to promote cholesterol homeostasis, a crucial process in injury-induced synaptic remodeling [191]. The daidzein glycoside puerarin (daidzein-8-C-glucoside), the main active compound of *Pueraria lobata,* has been extensively investigated for its beneficial pharmacological properties [193]. The compound was found to express protective effects in different cellular and animal models of stroke, including NHIB [194], brain ischemia [195,196,197,198,199,200] and SAH [201]. Puerarin protection may be mediated by its antiapoptotic and antioxidant activity through the modulation of factors and signaling pathways such as SOD, PI3K/Akt, MAPK and NF-κB [193].

Biochanin A, a natural isoflavonoid phytoestrogen derived from red clover or chickpea, displays a broad range of pharmacological functions, including neuroprotective activities [202]. The molecule was effective in alleviating brain damage and symptoms of rodents subjected to tMCAO [203,204] and SAH [205]. The activation of the protective factors glutamate oxaloacetate transaminase (GOT), SOD and Nrf2 and the inhibition of the NF-kB pathway may contribute to the neuroprotective effects of biochanin A [203,204,205].

#### 5.1.6. Anthocyanins

Anthocyanins are strongly pigmented compounds present in brightly colored fruits and vegetables. For their multiple pharmacological properties, anthocyanins have been investigated for the prevention or treatment of different diseases, including stroke [206].

The cyanidin glycoside cyanidin-3-O-glucoside is one of the most common anthocyanins and accounts for >95% of the total anthocyanin in Chinese bayberry (*Myrica rubra*) [207]. Recent findings pointed out a protective activity of cyanidin-3-O-glucoside in a mouse model of brain ischemia through the modulation of TLR4, NF-κB, Nrf2 and NLPR3 [208]. Similarly, cyanidin-3-O-glycosides protected PC12 cells against OGD-induced injury and provided beneficial effects in rodent models of brain ischemia by modulating antioxidant factors such as HO-1 [209,210,211]. Furthermore, cyanidin promoted protection in pial microcirculation of a rat model of global ischemia [212]. The protective effects on BBB integrity were mediated by arteriolar vasodilation via NO release and reduction of ROS levels [212].

#### 5.1.7. Dihydrochalcones

A few dihydrochalcones, a family of bicyclic flavonoids, have shown antioxidant properties [213]. In particular, phloretin, a dihydrochalcone abundant in apples and apple-derived products, displayed a neuroprotective effect by activating the Nrf2 pathway in rats subjected to tMCAO [214].

#### 5.1.8. Proanthocyanidins

Proanthocyanidins are oligomers or polymers of monomeric flavan-3-ols, particularly catechin and epicatechin. They are present in several plants, including apples, pine barks, cinnamon, blueberry and green and black tea. Proanthocyanidins are known for their cardioprotective and neuroprotective properties [215] and have also been investigated in stroke models.

For example, grape seed proanthocyanidin extract (GSPE) pretreatment alleviated brain damage in an NHIBI model, possibly through its antiapoptotic activity [216].

Procyanidins, composed of (-)-epicatechin units, significantly attenuated BBB disruption and neurological deficits in rodent brain ischemia models [217,218]. The neuroprotection was paralleled by a reduction of apoptosis and oxidative stress and an increase in angiogenesis [217,218]. Of note, apple polyphenols, which contains approximately 64% of procyanidins, prevented the formation of cerebral vasospasm in a rabbit model of SAH [219], suggesting a possible role for procyanidins in the treatment of this disease.

### 5.2. Phenolic Acids

#### 5.2.1. Hydroxycinnamic Acids

Hydroxycinnamic acids are a group of dietary phenolic compounds derivatives of cinnamic acid found abundant in cereals, legumes, oilseeds, vegetables and various beverages. They are present as four basic molecules, namely caffeic acid, ferulic acid, sinapic acid and p-coumaric acid [220].

Caffeic acid is found in thyme, sage, spearmint, sunflower seeds, yerba mate, coffee, wine, olive oil and various spices. Its derivative caffeic acid phenethyl ester (CAPE) is an active component of propolis. Thanks to their antioxidant, anti-inflammatory, cardioprotective and neuroprotective activities, these compounds have been investigated as antistroke agents [221,222]. CAPE administered either before or after ischemia reduced neonatal brain injury in an NHIBI rat model by inhibiting apoptosis and inflammation [223]. Pre- or postischemia administration of caffeic acid and CAPE also exerted protective effects in mature brains, improving outcomes in various in vivo models of brain ischemia [224,225,226,227,228]. The neuroprotection provided by these molecules was likely to be mediated through their antioxidant and anti-inflammatory actions and the inhibition of 5-lipoxygenase [224,225,226,227,228].

Ferulic acid is commonly present in the leaves, fruits and seeds of many plants such as rice, wheat, oats and giant fennel. The molecule is known for its multiple biological activities, including antioxidant, anti-inflammatory and antithrombotic actions [229]. Ferulic acid exerted protective effects in a cellular model of brain ischemia as well as in animal models of global and focal cerebral ischemia [230,231,232,233,234]. The neuroprotective effect of ferulic acid could be mediated by the anti-inflammatory and neurotrophic actions promoted by the reduction of intercellular adhesion molecule-1 (ICAM-1) and increase in brain levels of erythropoietin (EPO) and granulocyte colony-stimulating factor (G-CSF) [232,233,234].

Sinapic acid and its derivatives are orally available compounds found in spices, citrus and berry fruits, vegetables and cereals [235]. Postischemia sinapic acid treatment reduced neuronal damage and memory deficits in the four-vessel occlusion (4VO) rat model of global cerebral ischemia [236].

The main sources of p-coumaric acid are tea, coffee, wine, beer and various vegetables and fruits. Many studies have shown the beneficial properties of p-coumaric acid, including neuroprotective and anti-inflammatory effects [237]. The compound promoted neuroprotection in animal models of focal [238] and global brain ischemia [239] by hampering ROS production and apoptosis.

Chlorogenic acid, a caffeic acid–quinate conjugate, is a major component of coffee, tea and several fruits or vegetables. Studies on chlorogenic acid suggest that it may promote neuroprotection against stroke through multiple effects [240]. Chlorogenic acid (and its metabolite dihydrocaffeic acid) administered either before or after ischemia reduced brain infarct volume, BBB damage and behavioral deficits in tMCAO rats by blunting MMP activation and increasing brain levels of EPO, HIF-1a and nerve growth factor (NGF) [240,241,242]. Moreover, the compound promoted neuroprotection in rats subjected to 2VO by regulating the Nrf2 pathway [243]. Of note, the combination of chlorogenic acid with rtPA was effective in reducing behavioral deficits in the rabbit SCE model, extending the therapeutic time window for rtPA administration [244].

Rosmarinic acid is a natural antioxidant hydroxycinnamate commonly found in Lamiaceae and Boraginaceae plant families, including rosemary, sage, basil, thyme and peppermint [245]. The compound protected SH-SY5Y cells against OGD-induced cell death [246,247]. Pre- or postischemia administration of rosmarinic acid alleviated brain injury and memory impairment in MCAO animal models through the modulation of Nrf2, HO-1 and synaptophysin [248,249]. Moreover, rosmarinic acid protected diabetic rats against ischemic assault by attenuating BBB breakdown even when administered 5 h after the stroke onset [247]. The protective effects of the molecule may involve an anti-inflammatory action through the modulation of high-mobility group box1 (HMGB1) and the NF-κB signaling pathway [247].

#### 5.2.2. Hydroxybenzoic Acids

Recent findings suggest that gallic acid, a benzoic acid found in tea leaves and red wine, could play a protective role against stroke [250]. Prestroke treatment with gallic acid promoted neuroprotection in cellular and animal models of cerebral ischemia [251,252]. Interestingly, administration of the molecule before stroke onset mitigated brain injury and behavioral deficits in a rat model of global brain ischemia exposed to particulate matter [253]. Furthermore, administration of gallic acid and its derivatives reduced depressive symptoms and oxidative stress in a mouse model of poststroke depression [254].

### 5.3. Stilbenes

Stilbenes are part of a vast group of natural defense compounds occurring in many plants. These molecules are endowed with a wide range of beneficial activities, including the capability to protect against oxidative stress [255].

Among stilbenes, resveratrol is by far the most widely studied for its beneficial properties [256,257]. Pre- or poststroke treatment with resveratrol or its glucoside derivative polydatin showed beneficial effects in a large number of models of stroke (for complete reviews, see [258,259]). Recent examples of stroke models that benefited from the actions of resveratrol include NHIBI models [260,261,262], cellular cultures challenged with OGD [263,264,265], rodents subjected to MCAO [264,266,267,268,269] and ICH and SAH rodent models [270,271,272,273,274]. The potential mechanisms of action underlying the effects of resveratrol against stroke are numerous since the molecule interacts with a wide range of enzymes and receptors and promotes the expression of several factors devoted to enhancing cellular stress resistance and reducing apoptosis [259]. It has been proposed that beneficial properties of resveratrol could be mediated by its modulatory action on sirtuins and AMP-activated kinase (AMPK), a serine/threonine kinase known to be a key metabolic and stress sensor/effector [65,264,266,267].

### 5.4. Curcuminoids

Curcuminoids consist of curcumin and its derivatives, molecules found in the rhizome of turmeric (*Curcuma longa*). Curcumin possesses several beneficial properties that could make it a suitable candidate for stroke prevention or treatment, including anti-inflammatory, antilipemic, antiaggregant, neuroprotective and epigenetic modulatory activities [275]. Pre- and posthypoxia treatment with curcumin was found to effectively promote neuroprotection in rat neurons challenged with OGD [276], in neonatal mice subjected to hypoxic–ischemic brain injury [277], in MCAO rodents [278,279,280,281,282,283,284] and in stroke-prone spontaneously hypertensive rats [285].

### 5.5. Lignans

Lignans are a large group of polyphenolic compounds present in good quantity in various plants, including flax and sesame seeds, and whole bran cereals [286]. Some dietary lignans have been suggested to have potential in the prevention of cardiovascular disease and, possibly, stroke [287,288].

For example, administration of pinoresinol, a lignan found in sesame feed, *Brassica* vegetables and olive oil, prevented pial circulation damage induced by 2VO in rats by reducing oxidative stress [289].

### 5.6. Ellagitannins and Ellagic Acid

Ellagitannins and ellagic acid are polyphenols present in different fruits, including pomegranates, strawberries, black raspberries, raspberries, walnuts and almonds. In vivo, ellagitannins are hydrolyzed to ellagic acids which in turn are metabolized by intestinal microbiota to different types of urolithins [46]. Some molecules of this polyphenol class show neuroprotective properties against stroke.

Ellagic acid protected against ischemic injury in both cellular and in vivo models of brain ischemia by regulating Bcl-2/Bax expression [290].

Among urolithins, urolithin A has been shown to mitigate OGD-induced damage in N2a neuroblastoma cells and primary neurons and to reduce ischemic brain injury in mice by inhibiting endoplasmic reticulum (ER) stress [291].

Punicalagin, a natural ellagitannin found at high concentration in pomegranates, reduced infarct volume and neurological deficits in rats subjected to tMCAO through its antioxidant, anti-inflammatory and antiapoptotic properties [292,293].

### 5.7. Coumarins

Coumarin and coumarins, its derivatives, can be found in many plants, including tonka beans (where they are present in high concentration), *Cinnamon cassia* and cherry blossom of the genus *Prunus* [294]. The use of coumarins in the prevention of ischemic stroke is based on the anticoagulant or antiplatelet effects exhibited by many of these molecules [295,296,297,298]. Recent findings indicate that coumarins, besides their cardiovascular effects, may promote neuroprotection in preclinical models of stroke.

For example, auraptene, a citrus coumarin endowed with anti-inflammatory properties [299], acts as neuroprotective agent in the 2VO mouse model of brain ischemia by inhibiting inflammation [300,301].

Umbelliferone, a natural coumarin derivative with antioxidant and scavenging properties [302], ameliorated neurological outcomes and brain injury in tMCAO rats partly through the inhibition of thioredoxin-interactive protein (TXNIP)/NLRP3 inflammasome and activation of peroxisome proliferator-activated receptor-γ (PPAR-γ) [303].

Esculetin, another natural coumarin compound studied for its antioxidant and anti-inflammatory activities [304], promoted neuroprotection in mice subjected to tMCAO via upregulation of Bcl-2 and downregulation of Bax, two proteins involved in apoptosis [305].

Imperatorin is a naturally occurring coumarin that over the years has gained increasing interest for its health properties [306]. The compound exerted a protective activity in the SH-SY5Y neuroblastoma cell line subjected to OGD and in a rat model of brain ischemia [307]. The beneficial effect of imperatorin was associated with a reduction of apoptosis and upregulation of BDNF [307].

Scopoletin, a coumarin compound used in traditional Chinese medicine, has been studied for its antioxidant and anti-inflammatory properties [308]. A recent study pointed out a neuroprotective effect of scopoletin in a rat model of brain ischemia [309].

Osthole is a natural coumarin derivative isolated in several medicinal plants [310]. Various studies support the neuroprotective abilities of osthole in in vivo models of brain ischemia by hampering apoptosis [311,312,313].

Similar to other coumarins, daphnetin, a natural compound extracted from medicinal herbs, has been reported to show multiple beneficial properties [302]. Daphnetin provided neuroprotection in animal models of cerebral ischemia in both immature and adult brains by reducing inflammatory cytokine production and neuronal apoptosis [314,315].

The polyphenols active in preclinical models of stroke, whether the treatment was started before, during or after stroke induction (pre, concomitant or post, respectively), effective polyphenol concentration/dose and length of the treatment, and polyphenol major effects are reported in Table 1.

## 6. Polyphenols and Stroke: Results from Human Studies

Clues about the relationship between dietary consumption of polyphenols and beneficial effects on human health come from epidemiological studies.

In general, the inverse association between intake of high-polyphenol content food (e.g., fruits and vegetables) and risk of stroke appears clear, even though the role of polyphenols in this protection is still debated [316]. Moreover, fruit-derived polyphenol supplementation has been shown to improve cognitive and functional recovery of ischemic stroke patients [317].

The association between the consumption of beverages rich in polyphenols (i.e., wine, beer, coffee and tea) and stroke has been investigated. Although alcohol consumption at high intakes is detrimental even when occasionally consumed, a moderate intake of wine and beer has been associated with a lower risk of cardiovascular disease and ischemic stroke (for a review, see [318]). The protective effects related to wine and beer consumption have been attributed not only to ethanol itself but also to nonalcoholic components, mainly polyphenols [319,320]. Moreover, moderate consumption of coffee has been suggested to reduce the incidence of cardiovascular diseases [321]. Conversely, the association between coffee intake and stroke is under debate, with some studies indicating the beneficial effect of this beverage and others showing positive or no associations [322]. Dose–response analyses of tea intake indicate that high consumption of green tea was related to a reduced risk of both ischemic and hemorrhagic stroke [323].

Another typically polyphenol-rich food is cocoa, mostly consumed as chocolate in Western countries. Although few prospective studies on chocolate and stroke exist, the available data suggest that chocolate consumption could reduce the risk of coronary heart disease and stroke [324].

An important source of polyphenols is represented by culinary spices and herbs that, besides their use in cooking to add flavor to food dishes, are also employed in traditional medicines to prevent or treat different conditions. The effectiveness of traditional herbal medicine in stroke prevention and treatment has been reported in a variety of preclinical and clinical studies. However, solid conclusions about the relationship between spices/herbs and the risk of stroke cannot be made due to the methodological gaps of many investigations [325].

Scientific evidence about the antistroke role of food containing specific polyphenol classes or isolated polyphenolic compounds is still scarce, although the results from the available studies are generally promising.

Results from a meta-analysis considering 11 prospective cohort studies suggested that high dietary intake of flavonoids may moderately reduce the risk of stroke [326].

Different prospective studies also examined the relationship between dietary flavonoid subclasses and stroke. Higher dietary flavonol intake has been associated with a reduced risk for stroke [327,328]. In a cohort study, the consumption of food rich in quercetin was associated with a decreased risk of thrombotic stroke [329]. The quercetin metabolite 4-methylcatechol displayed a relevant antiplatelet activity in human blood, supporting a possible use of this molecule in the prevention of thrombotic stroke [330]. Moreover, a meta-analysis of randomized controlled trials indicated a significant effect of quercetin supplementation in reducing blood pressure [331]. In a double-blind randomized clinical trial among stroke patients, fisetin was found to prolong the therapy window of rtPA treatment, likely by reducing levels of MMPs and C-reactive protein (CRP) [332].

A similar effect in extending the rtPA therapy window was observed in a clinical trial involving the use of EGCG [333]. In this study, the beneficial effect of the polyphenolic compound could also be attributed to the reduction of plasma levels of MMPs [333]. The finding that pyrogallol, a human metabolite of EGCG from green tea, inhibited platelet aggregation in human blood [330] suggests that inhibition of platelet formation could play a role in the protective effect of green tea against brain ischemia.

The increased intake of flavanones has been associated with diminished risk of brain ischemia in women [334]. The flavanone pinocembrin has been approved by the China Food and Drug Administration for the treatment of ischemic stroke, and it is currently under phase II clinical trial [132].

In an observational study, administration of luteolin in combination with the lipid amide palmitoylethanolamide (PEA) promoted clinical improvement in stroke patients, when compared with literature data of patients with similar pathological conditions that did not receive pharmacological treatment [335].

The frequent intake of soybeans and soy food like soymilk or tofu, containing high levels of genistein and other isoflavones, has been associated with a reduced risk of stroke in Japanese and Chinese populations [336,337]. The antiplatelet potential of many isoflavones might contribute to the cerebrovascular protection provided by a soy-rich diet. In support of this, the isoflavones genistein and tectorigenin showed a strong antiplatelet effect when tested in human blood [53]. The isoflavone puerarin is an important component of traditional Chinese medicine. A recent meta-analysis of 35 randomized controlled trials on the effect of puerarin injections in acute cerebral ischemia suggests a possible clinical use of the compound against stroke [338]. However, due to the poor methodological quality of some of the studies, further clinical trials are needed to verify the safety of the drug [338].

Different clinical studies have indicated that consumption of food rich in anthocyanins, including blueberries and cranberries, improves cerebral blood flow in healthy adults (for a review, see [206]). However, a recent meta-study on 19 prospective cohorts demonstrated that there was no relationship between consumption of anthocyanins and different types of strokes, although that dietary intake of anthocyanins was inversely correlated with the risk of cardiovascular diseases [339].

Some recent cohort studies indicated an inverse association between phenolic acids (hydroxybenzoic and hydroxycinnamic acids in particular) and cardiovascular diseases and hypertension (for a recent review, see [340]). Different clinical trials confirmed a moderate effect of chlorogenic acid in reducing blood pressure in mild hypertensive adults [341]. To our knowledge, no clinical studies are available on the correlation between phenolic acids and stroke.

Long-term resveratrol supplementation in patients who suffered a stroke in the previous 12 months promoted a beneficial effect on blood pressure, body mass index and lipid profile, indicating a possible role of resveratrol as an adjuvant in the secondary prevention of stroke [342]. Moreover, resveratrol administration improved the outcomes of stroke patients receiving rtPA, suggesting that this polyphenol could serve as a potential adjuvant of rtPA therapies [343]. A positive correlation between resveratrol-promoted outcomes and reduction of plasma levels of MMPs was observed [343].

## 7. Conclusions

The incomplete list of polyphenols active in cellular and animal models presented here strongly supports the potential role of many classes of polyphenols against different types of stroke. Interestingly, many molecules also exert beneficial effects when administered after the stroke onset, suggesting that they could be exploited in the treatment of this pathology. Of note, some compounds were able to synergize with rtPA, indicating a possible use as coadjuvant in the current treatment of stroke.

Notwithstanding the body of positive preclinical findings, conclusive evidence from human studies is still lacking. Although the consumption of food rich in polyphenols is generally associated with positive health effects, including a lower incidence of cardiovascular disease and stroke, the effectiveness of isolated polyphenols in stroke treatment is still under debate.

Individuals consume polyphenols not as isolated compounds but rather as components of their overall daily diet. The variability in food composition and the difficulty in determining the accurate quantity of polyphenols in food, the potential modulation by food matrices and culinary techniques, the possible interactions between polyphenols with each other or with other food components and the effect of gut microbiota metabolism on the bioavailability of polyphenols, make studies in human population extremely challenging. In the future, long-term, large-scale and well-designed clinical trials are required to establish the effectiveness of the most promising polyphenols that have emerged from studies on preclinical models of stroke.

Finally, it will be important to further characterize the molecular targets of polyphenolic compounds potentially active against stroke. The identification of common molecular pathways targeted by different bioactive components may lead to the formulation of novel nutritional supplements, where different polyphenols could synergize at doses much lower than those of active individual compounds.

## Figures and Tables

**Table 1:** Polyphenols active in preclinical stroke models.

Polyphenol Class	Polyphenol		Type of Stroke	Experimental Model	Effective Dose and Treatment	Findings	References
Flavonols	Quercetin		Ischemic Stroke	tMCAO rat model	Post; 20 mg/kg; daily for 3 days Pre; 25 mg/kg; daily for 21 days	↓brain damage, oxidative stress, apoptosis, neurological deficits	[69,70]
			Ischemic Stroke	pMCAO rat model	Post; 30 mg/kg; single administration	↓brain damage	[71]
			Ischemic Stroke	Photothrombotic rat model	Post; 25 µmol/kg; every 12 h for 3 days Post; 50 mg/kg; every 12 h for 3 days	↓BBB damage, neurological deficits	[72,73]
			Ischemic Stroke	2VO mouse model	Pre; 50 mg/kg; 30 min before and immediately after stroke, then daily for 2 days	↓ brain damage, BBB damage	[74]
			ICH	Collagenase infusion rat model	Post; 30 mg/kg; single administration Post; 50 mg/kg; single administration	↓brain damage, oxidative stress, apoptosis, inflammation, neurological deficits	[75,76]
			SAH	Blood infusion rat model	Post; 10 mg/kg; every 8 h for 2 days Post; 50 mg/kg; 30 min, 12 h and 24 h after SAH	↓brain damage, oxidative stress, vasospasm, apoptosis, neurological deficits	[77,78]
	Myricetin		Ischemic Stroke	OGD SH-SY5Y cells	Pre; 0.1 nM	↓toxicity	[80]
			Ischemic Stroke	OGD human BMVECs	Pre; 30 µM	↓oxidative stress, inflammation, endothelial permeability	[82]
			Ischemic Stroke	tMCAO rat model	Pre; 20 mg/kg; 2 h before and daily for 2 days after stroke Pre; 25 mg/kg; daily for 7 days	↓brain damage, oxidative stress, apoptosis, inflammation, neurological deficits	[80,81]
	Kaempferol		Ischemic Stroke	tMCAO rat model	Pre; 100 and 200 µM; 30 min before and immediately after reperfusion Post; 10 and 7.5 mg/kg; single administration Post; 100 mg/kg; daily for 7 days	↓brain damage, oxidative stress, apoptosis, inflammation, BBB damage, neurological deficits	[84,85,86]
	Fisetin		Ischemic Stroke	tMCAO mouse model	Pre; 50 mg/kg; single administration Post; 50 mg/kg; single administration	↓brain damage, inflammation	[89]
			Ischemic Stroke	pMCAO rat model	Post; 30 mg/kg; single administration	↓brain damage	[71]
			Ischemic Stroke	SCE rabbit model	Post; 50 mg/kg; single administration	↓ neurological deficits	[88]
	Morin		Ischemic Stroke	tMCAO rat model	Post; 30 mg/kg; daily for 7 days Post; 30 mg/kg; single administration	↓brain damage, BBB damage, inflammation, oxidative stress, apoptosis, neurological deficits	[91,92]
Flavan-3-ols	EGCG		Ischemic Stroke	OGD PC12 cells	Pre; 2 µM	↓toxicity, apoptosis	[97]
			Ischemic Stroke	tMCAO mouse model	Post; 50 mg/kg; single administration	↓brain damage, MPPs	[98]
			Ischemic Stroke	tMCAO rat model	Post; 50 mg/kg; single administration Post; 50 mg/kg; single administration Post; 50 mg/kg; daily for 3 days Post; 50 mg/kg; single administration Post; 20 mg/kg; single administration Pre; 40 mg/kg; daily for 3 days	↓brain damage, oxidative stress, apoptosis, inflammation, neurological deficits	[99,100,101,102,103,104]
			Ischemic Stroke	2VO gerbil model	Post; 25 and 30 mg/kg; single administration Pre; 50 mg/kg; 30 min before and immediately after stroke	↓brain damage, oxidative stress	[105,106]
			Ischemic Stroke	tMCAO rat model treated with rtPA	Post; 20 mg/kg; single administration	↓brain damage, BBB damage, neurological deficits	[107]
			SAH	Hb PC12 cells	Pre; 1–50 µM Pre; 1–50 µM Pre, 50 µM	↓cell proliferation	[108,109,110]
			SAH	Hb mouse model	Pre; 50 mg/kg; daily for 14 days Pre; 50 mg/kg; daily for 14 days	↓brain damage, oxidative stress, mitochondrial dysfunction, apoptosis, neurological deficits	[109,110]
	ECG		Ischemic Stroke	OGD PC12 cells	Pre; 2 µM	↓toxicity, apoptosis	[97]
			Ischemic Stroke	OGD human BMVECs	Pre; 2 µM	↓oxidative stress, apoptosis	[111]
	EC		Ischemic Stroke	OGD mouse neurons	Pre; 100 µM	↓toxicity, oxidative stress	[113]
			Ischemic Stroke	tMCAO mouse model	Pre; 30 mg/kg; single administration Post; 30 mg/kg; single administration	↓brain damage, neurological deficits	[112]
			Ischemic Stroke	pMCAO mouse model	Pre; 15 mg/kg; single administration	↓brain damage, inflammation	[113]
			ICH	Hb mouse astrocytes	Pre; 100 µM	↓oxidative stress	[114]
			ICH	Collagenase infusion mouse model	Post; 15 mg/kg; 3 h after ICH and daily for 3 days	↓brain damage, oxidative stress, neurological deficits	[115]
			ICH	Blood infusion mouse model	Post; 15 mg/kg; 3 h after ICH and daily for 3 days	↓brain damage, oxidative stress, neurological deficits	[115]
			ICH	Thrombin infusion mouse model	Post; 15 mg/kg; 3 h after ICH and daily for 3 days	↓brain damage, oxidative stress, neurological deficits	[115]
Flavanones	Eriodictyol		Ischemic Stroke	OGD rat astrocytes	Pre; 20–80 µM	↓toxicity	[120]
			Ischemic Stroke	tMCAO rat model	Pre; 30 mg/kg; daily for 5 days	↓brain damage, neurological deficits	[120]
			Ischemic Stroke	pMCAO mouse model	Pre; 4 mg/kg; 30 min before, 2 h and daily for 5 days after stroke	↓brain damage, inflammation, neurological and memory deficits	[119]
	Naringenin		Ischemic Stroke	OGD rat neurons	Pre; 80 µM Post; 80 µM	↓toxicity, oxidative stress, apoptosis, mitochondrial dysfunction	[122,123]
			Ischemic Stroke	OGD human MSCs	Post; 40 and 80 µM	↓ inflammation	[124]
			Ischemic Stroke	pMACO rat model	Pre; 100 mg/kg; daily for 4 days	↓brain damage, inflammation, neurological deficits	[126]
			Ischemic Stroke	tMCAO rat model	Post; 80 µM; single administration Pre; 50 mg/kg; daily for 21 days	↓brain damage, oxidative stress, apoptosis, inflammation, neurological deficits	[123,125]
			Ischemic Stroke	Stroke-prone spontaneously hypertensive rats	9.0 and 17.7 mg/kg; daily for 4 weeks	↓blood pressure, thrombotic tendency, oxidative stress	[127]
	Hesperetin		Ischemic Stroke	tMCAO rat model	Pre; 50 mg/kg; daily for 15 days	↓brain damage, oxidative stress, inflammation, neurological and behavioral deficits	[129]
			Ischemic Stroke	2VO rat model	Pre; 20 mg/kg; daily for 14 days	↓brain damage, oxidative stress, inflammation	[130]
			Ischemic Stroke	Stroke-prone spontaneously hypertensive rats	14.5 (hesperidin), 16.2 and 31.6 (glucosyl hesperidin) mg/kg; daily for 4 weeks	↓blood pressure, thrombotic tendency, oxidative stress	[127]
			SAH	Blood infusion rat model	Post; 50 and 100 mg/kg; daily for 2 days	↓vessel wall thickness, ↑vessel luminal diameter	[131]
	Pinocembrin		Ischemic Stroke	OGD rat neurons	Post; 0.1, 1 and 10 µM	↓toxicity, oxidative stress, apoptosis	[133]
			Ischemic Stroke	OGD rat BMVECs	Post; 1 and 10 µM	↓toxicity	[134]
			Ischemic Stroke	OGD human BMECs and astrocytes	Post; 1 µM	↓endothelial permeability	[135]
			Ischemic Stroke	tMCAO rat model	Post; 3–30 mg/kg; single administration Post; 1–10 mg/kg; single administration	↓brain damage, oxidative stress, apoptosis, neurological deficits	[133,136]
			Ischemic Stroke	pMCAO rat model	Post; 3, 10 and 30 mg/kg; single administration	↓brain damage, apoptosis, inflammation, neurological deficits	[137]
			Ischemic Stroke	2VO rat model	Pre; 10 mg/kg; daily for 7 days	↓brain damage, oxidative stress, apoptosis, inflammation	[138]
			Ischemic Stroke	4VO rat model	Post; 1–10 mg/kg; single administration	↓brain and BBB damage, neurological deficits	[134]
			Ischemic Stroke	Thromboembolic rat model treated with rtPA	Post; 10 mg/kg; single administration or daily for 7 days	↓brain and BBB damage, neurological deficits	[135]
			ICH	Collagenase infusion mouse model	Post; 5 mg/kg; 2 h after ICH and every 12 h for 3 days	↓brain damage, inflammation, neurological deficits	[139]
Flavones	Luteolin		NHIB	OGD rat neurons	Pre; 10–30 µM	↓toxicity, oxidative stress, apoptosis	[142]
			Ischemic Stroke	OGD mouse neurons	Post; 0.1–1 µM	↓toxicity	[143]
			Ischemic Stroke	OGD mouse MCs	Pre; 10 and 100 nM	↓MC degranulation	[143]
			Ischemic Stroke	OGD human BMVECs	Post; 90 µM	↓toxicity, apoptosis	[144]
			Ischemic Stroke	tMCAO rat model	Post; 20–80 mg/kg; 0 and 12 h after stroke	↓brain damage, inflammation, neurological deficits	[145]
			Ischemic Stroke	pMCAO rat model	Post; 10 and 25 mg/kg; single administration Post; 10 and 25 mg/kg; single administration Post; 10 and 25 mg/kg; 0 h and daily for 3 days after stroke	↓brain damage, oxidative stress, apoptosis, neurological deficits	[144,146,147]
			ICH	Hb rat neurons	Post; 10 µM	↓toxicity	[148]
			ICH	Hb rat neurons and microglia	Post; 10 and 20 µM	↓ microglia activation and induced neurotoxicity	[149]
			ICH	Blood infusion rat model	Post; 10 and 20 mg/kg; single administration Post; 10 and 20 mg/kg; 30 min, 12 h and 24 h after ICH	↓brain damage, inflammation, neurological and memory deficits	[148,149]
	Apigenin		Ischemic Stroke	OGD human BMVECs	Pre; 5 µM	↓toxicity, apoptosis, autophagy	[151]
			Ischemic Stroke	tMCAO rat model	Post; 25 mg/kg; daily for 7 or 14 days Post; 20 and 40 mg/kg; daily for 28 days	↓brain damage, neurological and memory deficits	[151,152]
			SAH	Endovascular perforation rat model	Post; 20 mg/kg; single administration	↓brain and BBB damage, inflammation, neurological deficits	[153]
	Nobiletin		Ischemic Stroke	Hypoxia HepG2 cells	Post; 100 µg/mL	↓toxicity	[160]
			Ischemic Stroke	tMCAO rat model	Post; 15 mg/kg; 0 and 1 h after stroke	↓brain damage, apoptosis, inflammation, neurological deficits	[157,160]
			Ischemic Stroke	2VO mouse model	Pre; 50 mg/kg; daily for 7 days before and 7 days after stroke	↓brain damage, memory deficits	[156]
	Tangeretin		Ischemic Stroke	OGD human BMVECs	Post; 2.5, 5 and 10 µM	↓toxicity, oxidative stress, apoptosis	[159]
			Ischemic Stroke	Hypoxia HepG2 cells	Post; 10 and 100 µg/mL	↓toxicity	[160]
	Baicalein		Ischemic Stroke	OGD SH-SY5Y cells	Pre; 0.1, 1 and 10 µM	↓toxicity	[163]
			Ischemic Stroke	tMCAO rat model	Post; 25 and 50 mg/kg; single administration Post; 50, 100 and 200 mg/kg; single administration	↓brain damage, oxidative stress, apoptosis, neurological deficits	[163,164]
			Ischemic Stroke	2VO gerbil model	Post; 100 and 200 mg/kg; single administration	↓brain damage, oxidative stress, apoptosis	[165]
	Acacetin		Ischemic Stroke	tMCAO mouse model	Post; 25 mg/kg; single administration	↓brain damage, inflammation, neurological deficits	[167]
Isoflavone	Genistein		Ischemic Stroke	OGD rat neurons	Concomitant, 1 mM	↓toxicity, apoptosis	[172]
			Ischemic Stroke	OGD PC12 cells	Concomitant, 30 µM	↓toxicity, apoptosis	[173]
			Ischemic Stroke	tMCAO mouse and rat models	Pre; 5 and 10 mg/kg; daily for 14 days Pre; 10 mg/kg; daily for 14 days Pre; 500 mg/kg (genistein), 250 mg/kg (equol); daily for 14 days Pre; 500 mg/kg; daily for 28 days before and 1 day after stroke Post; 10 mg/kg; daily for 3 days Post; 1 and 2 mg/kg; single administration Pre; 10 mg/kg; daily for 14 days	↓brain damage, oxidative stress, apoptosis, neurological deficits ↑circulatory function	[174,175,176,177,178,179,180]
			Ischemic Stroke	pMCAO rat model	Post; 10 mg/kg; single administration	↓brain damage, oxidative stress, apoptosis, neurological deficits	[181]
			Ischemic Stroke	Photothrombotic rat model	Pre; 16 mg/kg; every 6 h from 24 h before to 24 h after stroke	↓brain and BBB damage	[182]
			Ischemic Stroke	2VO mouse model treated with streptozotocin	Pre; 5 and 10 mg/kg; daily for 14 days before and 1 day after stroke	↓brain damage, oxidative stress, apoptosis, neurological deficits	[183]
			Ischemic Stroke	4VO rat model	Pre; 0.1 mg/kg; daily for 7 days before and 7 days after stroke Post; 1 mg/kg; single administration Pre; 15 mg/kg; 30 min before and 24 h after stroke	↓brain damage, oxidative stress, apoptosis, neurological deficits	[184,185,186]
			Ischemic Stroke	2VO gerbil model	Post; 3 and 10 mg/kg; single administration	↓brain damage, oxidative stress, behavioral and memory deficits	[187]
			SAH	Blood infusion rat and dog models	Post; 10 µM; single administration Post; 14.0 and 17.2 µM; single administration	↓vasospasm	[188,189]
	Daidzein		NHIBI	OD rat NSCs	Pre; 20–100 µM	↓toxicity, apoptosis	[194]
			Ischemic Stroke	OGD rat astrocytes	Concomitant, 12 and 48 µg/mL	↓toxicity, apoptosis	[195]
			Ischemic Stroke	tMCAO mouse and rat models	Post; 10 mg/kg; daily for 7 days, then every other day up to 1 month Pre; 50 and 100 mg/kg; daily for 7 days Pre; 36 and 54 mg/kg; daily for 5 days Pre; 50 and 100 mg/kg; single administration Post; 50 and 100 mg/kg; daily for 14 days	↑cholesterol homeostasis, ↓brain damage, inflammation, neurological, motor and memory deficits	[191,196,197,198,199]
			Ischemic Stroke	pMCAO mouse and rat models	Post; 0.10 mg/kg; daily for 14 days Post; 10 mg/kg; single administration	↓brain damage, oxidative stress, apoptosis, behavioral deficits	[190,192]
			Ischemic Stroke	Stroke-prone spontaneously hypertensive rats	100 mg/kg; daily for 14 days	↓arterial dysfunction, blood pressure	[200]
			SAH	Endovascular perforation mouse model	Pre; 100 mg/kg; single administration	↓brain and BBB damage, oxidative stress, apoptosis, neurological deficits	[201]
	Biochanin A		Ischemic Stroke	tMCAO mouse and rat models	Pre; 5 and 10 mg/kg; daily for 4 weeks Pre; 10, 20 and 40 mg/kg; daily for 14 days	↓brain damage, oxidative stress, neurological deficits	[203,204]
			SAH	Blood infusion rat model	Post; 20 and 40 mg/kg; single administration	↓brain damage, apoptosis, inflammation, neurological and memory deficits	[205]
Anthocyanins	Cyanidin		Ischemic Stroke	OGD PC12 cells	Concomitant, 10 µg/mL	↓toxicity	[209]
			Ischemic Stroke	tMCAO mouse model	Post; 150 and 300 mg/kg; daily for 7 days Post; 10 mg/kg; single administration	↓brain damage, oxidative stress	[208,209]
			Ischemic Stroke	2VO rat model	Pre; 10 mg/kg; 1 h before stroke/post; 10 mg/kg; 0.5 h after reperfusion Pre; 10 and 20 mg/kg; 10 min before stroke and during reperfusion	↓brain damage, oxidative stress, microvascular permeability	[210,212]
			Ischemic Stroke	pMCAO mouse model	Pre; 2 and 5 mg/kg; 1 h before stroke/post; 2 mg/kg; 3 h after stroke	↓brain damage, oxidative stress, neurological deficits	[211]
	Phloretin		Ischemic Stroke	tMCAO rat model	Pre; 40 and 80 mg/kg; daily for 14 days	↓brain damage, oxidative stress, neurological deficits	[214]
Dihydrochalcones	GSPE		NHIBI	Carotid ligation mouse model	Pre; 30 mg/kg; single administration	↓brain damage, apoptosis, neurological deficits	[216]
Proanthocyanidins	Procyanidins		Ischemic Stroke	tMCAO mouse, rat models	Post; 20 and 40 mg/kg; daily for 2–14 days Pre; 50 mg/kg; daily for 14 days	↓brain and BBB damage, oxidative stress, apoptosis, neurological deficits	[217,218]
			SAH	Blood infusion rabbit model	Post; 10 and 50 mg/kg; daily for 3 days	↓vasospasm	[219]
Hydroxycinnamic acids	Caffeic acid		NHIBI	Carotid ligation mouse model	Pre; 40 mg/kg; 30 min before and daily for 7 days after stroke/post; 40 mg/kg; single administration	↓brain damage, apoptosis, inflammation	[223]
			Ischemic Stroke	tMCAO rat model	Pre; 50 mg/kg; 30 min before, 0 h, 1 h and 2 h after stroke, then every 12 h for 4 days Pre; 0.1–10 µg/kg; single administration	↓brain damage, inflammation, neurological deficits	[224,227]
			Ischemic Stroke	pMCAO rabbit model	Pre; 10 µmol/kg; daily for 7 days	↓brain damage, oxidative stress, neurological deficits	[226]
			Ischemic Stroke	Photothrombotic mouse model	Post; 2 and 5 mg/kg; 1 and 6 h after stroke	↓brain damage, inflammation	[225]
			Ischemic Stroke	2VO rat model with hypotension	Post; 10, 30 and 50 mg/kg; single administration	↓brain damage, oxidative stress, memory deficits	[228]
	Ferulic acid		Ischemic Stroke	OGD PC12 cells	Pre; 80 and 100 µM	↓toxicity, oxidative stress	[230]
			Ischemic Stroke	tMCAO rat model	Post; 28, 56 and 112 mg/kg; daily for 5 days Post; 100 and 200 mg/kg; daily for 7 days Concomitant; 100 mg/kg; single administration Concomitant, 80 and 100 mg/kg/post; 100 mg/kg; single administrations	↓brain damage, oxidative stress, apoptosis, inflammation, neurological and memory deficits	[231,232,233,234]
			Ischemic Stroke	4VO rat model	Pre; 20 and 25 mg/kg; daily 4 days before, immediately after stroke and during reperfusion	↓brain damage, oxidative stress, neurological and memory deficits	[230]
	Sinapic acid		Ischemic Stroke	4VO rat model	Post; 3 and 10 mg/kg; 0 and 90 min after stroke or daily for 14 days	↓brain damage, memory deficits	[236]
	p-Coumaric acid		Ischemic Stroke	pMCAO rat model	Post; 100 mg/kg; single administration	↓brain damage, oxidative stress, apoptosis, neurological deficits	[238]
			Ischemic Stroke	2VO mouse model	Pre; 100 mg/kg; daily for 14 days	↓brain damage, oxidative stress	[239]
	Chlorogenic acid		Ischemic Stroke	tMCAO rat model	Post; 30 mg/kg; 0 and 2 h after stroke Post; 30 mg/kg; 0 and 2 h after stroke Pre; 15, 30 and 60 mg/kg; daily for 7 days	↓brain and BBB damage, oxidative stress, MMPs level, behavioral deficits	[240,241,242]
			Ischemic Stroke	2VO rat model	Post, 100 and 500 mg/kg	↓brain damage, oxidative stress, apoptosis, MMPs level, memory deficits	[243]
			Ischemic Stroke	SCE rabbit model treated with rtPA	Post; 50 mg/kg; single administration	↓behavioral deficits	[244]
	Rosmarinic acid		Ischemic Stroke	OGD SH-SY5Y cells	Pre; 1 and 10 µM Post; 3–81 µM	↓toxicity, apoptosis	[246,247]
			Ischemic Stroke	tMCAO mouse model	Post; 20 and 40 mg/kg; single administration	↓brain damage, oxidative stress, apoptosis	[248]
			Ischemic Stroke	tMCAO rat model treated with streptozotocin	Post; 50 mg/kg; single administration	↓brain and BBB damage, neurological deficits	[247]
			Ischemic Stroke	pMCAO mouse model	Pre; 1 and 20 mg/kg; 30 min before, 1 h after stroke and then daily for 5 days	↓brain damage, inflammation, neurological and memory deficits	[249]
Hydroxybenzoicacids	Gallic acid		Ischemic Stroke	OGD rat neurons	Concomitant, 50 µM	↓toxicity	[251]
			Ischemic Stroke	tMCAO rat model	Pre; 50 mg/kg; daily for 7 days Pre; 50 mg/kg; single administration	↓brain damage, oxidative stress, apoptosis, inflammation, mitochondrial dysfunction, neurological deficits	[251,252]
			Ischemic Stroke	4VO rat model exposed to particulate matter	Pre; 100 mg/kg; daily for 10 days	↓BBB damage, oxidative stress, behavioral deficits	[253]
			Ischemic Stroke	2VO mouse model	Post; 25 and 50 mg/kg; daily for 7 days	↓oxidative stress, depressive symptoms	[254]
Stilbenes	Resveratrol		NHIBI	Carotid ligation mouse and rat models	Post; 100 mg/kg; 0, 8 and 18 h after stroke Pre; 0.2 and 20 mg/kg; single administration/post; 20 mg/kg; single administration Pre; 20 mg/kg; single administration	↓brain damage, apoptosis, inflammation, behavioral deficits	[260,261,262]
			Ischemic Stroke	OGD mouse and rat neurons, PC12 cells	Pre, post, pre and post; 5–25 µM Post; 30 µM Pre and post, post; 10–80 µM Pre; 40 µM	↓toxicity, oxidative stress, apoptosis	[263,264,265,268]
			Ischemic Stroke	tMCAO mouse, rat models	Post; 6.8 mg/kg; single administration Post; 6.8 mg/kg; single administration Pre; 20 and 30 mg/kg; daily for 5 days Pre; 30 mg/kg; daily for 7 days and 30 min before stroke Post; 1.9 mg/kg; single administration	↓brain and BBB damage, apoptosis, neurological deficits	[264,266,267,268,269]
			ICH	Collagenase infusion mouse model	Post; 10 mg/kg; single administration	↓brain damage, apoptosis, inflammation, neurological deficits	[270]
			SAH	Blood infusion rat model	Post; 10 mg/kg; daily for 3 days Post; 60 mg/kg; 2 and 24 h after SAH	↓vasospasm, apoptosis	[271,272]
			SAH	Endovascular perforation rat model	Post; 30 mg/kg; 0 and 6 h after SAH Pre; 100 mg/kg; single administration	↓brain and BBB damage, apoptosis, neurological deficits	[273,274]
Curcuminoids	Curcumin		NHIBI	Carotid ligation mouse model	Pre; 100 µg/kg; single administration/post; 50–200 µg/kg; single administration	↓ brain damage, oxidative stress, apoptosis, inflammation	[277]
			Ischemic Stroke	OGD rat neurons	Pre; 0.5–8 µM	↓toxicity, apoptosis, inflammation	[276]
			Ischemic Stroke	tMCAO rat model	Post; 100 and 300 mg/kg; single administration Post; 300 mg/kg; single administration Post; 300 mg/kg; single administration Post; 300 mg/kg; daily for 7 days Post; 100, 300 and 500 mg/kg; single administration	↓brain damage, oxidative stress, apoptosis, inflammation, neurological deficits	[278,279,280,281,282]
			Ischemic Stroke	pMCAO mouse and rat models	Post; 50 mg/kg; single administration Post; 150 mg/kg; 0 and 24 h after stroke	↓brain damage, inflammation, neurological and behavioral deficits	[283,284]
			Ischemic Stroke	Stroke-prone spontaneously hypertensive rats	100 mg/kg; daily for 4 weeks	↓arterial dysfunction, oxidative stress, ↑survival	[285]
Lignans	Pinoresinol		Ischemic Stroke	2VO rat model	Pre; 1 and 2 mg/kg; 10 min before stroke and during reperfusion	↓microvascular damage, oxidative stress	[289]
Ellagitannins and ellagic acid	Ellagic acid		Ischemic Stroke	OGD rat neurons	Post; 10 and 30 µM	↓ toxicity, apoptosis	[290]
			Ischemic Stroke	Photothrombotic rat model	Pre; 10 and 30 mg/kg; 24 h before and immediately after stroke	↓neurological deficits	[290]
	Urolithin A		Ischemic Stroke	OGD mouse neurons and N2a cells	Pre; 3–30 µM	↓ toxicity	[291]
			Ischemic Stroke	tMCAO mouse model	Pre; 2.5 and 5.0 mg/kg; 24 and 1 h before stroke	↓ brain damage, neurological deficits	[291]
	Punicalagin		Ischemic Stroke	tMCAO rat model	Pre; 15 and 30 mg/kg; daily for 7 days Pre; 15 and 30 mg/kg; daily for 7 days	↓brain damage, oxidative stress, apoptosis, inflammation, neurological deficits	[292,293]
Coumarins	Auraptene		Ischemic Stroke	2VO mouse model	Post; 25 mg/kg; daily for 8 days Pre; 10 and 25 mg/kg; daily for 5 days	↓brain damage, inflammation	[300,301]
	Umbelliferone		Ischemic Stroke	tMCAO rat model	Pre; 15 and 30 mg/kg; daily for 7 days	↓brain damage, oxidative stress, inflammation, neurological deficits	[303]
	Esculetin		Ischemic Stroke	tMCAO mouse model	Pre; 50 and 100 mg/kg; single administration/post; 100 mg/kg; single administration	↓brain damage, apoptosis, neurological deficits	[305]
	Imperatorin		Ischemic Stroke	OGD SH-SY5Y cells	Concomitant, 2.56 µM	↓apoptosis	[307]
			Ischemic Stroke	tMCAO rat model	Pre; 10 mg/kg; single administration	↓brain damage, neurological deficits	[307]
	Scopoletin		Ischemic Stroke	tMCAO rat model	Pre; 1 mg/kg; single administration	↓brain damage	[309]
	Osthole		Ischemic Stroke	tMCAO rat model	Pre; 100 mg/kg; 30 min before stroke and immediately after reperfusion Pre; 40 mg/kg; single administration Pre; 20 and 40 mg/kg; single administration	↓brain damage, oxidative stress, apoptosis, MMPs levels, neurological deficits	[311,312,313]
	Daphnetin		NHIBI	Carotid ligation rat model	Pre; 10 mg/kg; single administration/post; 10 mg/kg; single administration	↓brain damage	[314]
			Ischemic Stroke	tMCAO mouse model	Pre; 1 mg/kg; single administration	↓brain damage, apoptosis, inflammation, neurological deficits	[314,315]

BMVECs: brain microvascular endothelial cells; EC: (−)-epicatechin; ECG: (−)-epicatechin-3-gallate; EGCG: epigallocatechin-3-gallate; GSPE: grape seed proanthocyanidin extract; Hb: hemoglobin; ICH: intracerebral hemorrhage; MCs: mast cells; MMPs: metalloproteinases; MSCs: mesenchymal stem cells; NHIBI: neonatal hypoxic–ischemic brain injury; NSCs: neural stem cells; OD: oxygen deprivation; OGD: oxygen and glucose deprivation; pMCAO: permanent middle cerebral artery occlusion; rtPA: recombinant tissue plasminogen activator; SAH: subarachnoid hemorrhage; SCE: small clot embolism; tMCAO: middle cerebral artery occlusion; 2VO: two-vessel occlusion; 4VO: four-vessel occlusion.

## Data Availability

No new data were created or analyzed in this study. Data sharing is not applicable to this article.
